# Dr. H. G. Davis’ Splint for Treatment of Morbus Coxarius

**Published:** 1862-12

**Authors:** 


### DR. H. G. DAVIS' SPLINT FOR TREATMENT OF MORBUS COXARIUS.

We have received from Dr. Davis a very beautifully constructed instrument for making extension and counter-extension in cases of morbus coxarius, also in disease of the knee joint. It consists of a hollow rod adapted to the side of the limb, at the upper extremity of which counter-extension is made by means of two perineal bands, one of which is elastic, and the other non-elastic, the former to yield to pressure, while the latter limits the extent of stretching. At the lower extremity of this rod is an ingenious arrangement for attaching it to bands of adhesive plaster, in such a manner that active extension can be made upon it. In the centre is an extending screw passing within the tube, by which any lengthening of the splint may be made which is desired, and thus extension and counter-extension fully accomplished, and at the same time the patient need not be confined, but can sit, walk and ride, with great convenience.

Dr. Davis claims the honor of originating the mode of treating joint disease by *continued elastic extension*, and there is no question that he is fully and clearly entitled to the credit of having introduced this method of treatment to the profession. It appears that the English have not yet said a word in reference to the originator, although some have adopted the principle. Barwell appropriates the credit of originating it, although Davis' instrument and its application had been known for months in England. We do not by this mean to be understood that he has *intentionally* failed to render honor where honor is due, but to claim the acknowledgment as an act of justice to American surgery, no less than to Dr. Henry G. Davis, of New York. In our own country we think his claims are almost unanimously conceded. As showing this fact we make a few extracts from remarks made by Dr. Alfred C. Post, chairman of a committee appointed by the section of surgery of the New York Academy of Medicine, for the purpose of examining into this subject, the other members, of which were Dr. Gurdon Buck and Prof. Willard Parker. After describing the instrument, he says:

"As long ago as 1850, Sir Benj. Brodie spoke of a means of treating morbus coxarius in an advanced stage, when shortening had taken place, by means of a cord attached to a band passing around the lower part of the thigh above the condyles. This passed over a pully at the foot of the bed, and had a weight attached to it for the purpose of keeping up continuous extension. This is the first instance of the application of what Dr. Davis calls *elastic extension* in the treatment of this disease. It is a principle, however, that has been known in the treatment of other diseases than the one under consideration, previous to this time. This mode of treatment differs, as can easily be seen, from the inelastic force which is applied to the limb when the ordinary straight splint is used. Brodie did not recommend this as a mode of treatment adapted to the disease in its different stages, or as the ordinary mode of treatment, but as a means of overcoming deformity; that which consists in shortening in its third stage. He spoke of it as a remedy of some value, but one which had disappointed him in most cases. Sir Benj. Brodie of course was not acquainted at that time with the mode of applying force in the extension of the limbs which has since been introduced by the use of adhesive plaster, which has proved itself so much superior to all other methods. All other methods for making extension are very faulty compared with this, and I have very little doubt that the obstacles which Brodie encountered were in consequence of the want of means.

During the last year a paper appeared in the *American Medical Monthly*, by Dr. H. G. Davis, giving an account of a method of treating morbus coxarius, which he had been in the habit of using for several years past, and his method is undoubtedly a very great improvement upon all others which have preceded it. We have, in the method described by Dr. Davis, the first intimation of extension being carried out in the treatment of this disease, *through all its stages*, in a manner which was calculated to relieve the sufferings of the patient, to arrest the progress of the disease, and at the same time to allow active exercise in the open air." * * *

"There is no question, Mr. President, that Dr. Davis is entitled to the credit of having introduced this method of treatment to the profession. It is true, at different periods, some one of these means has been employed by different surgeons; extension and counter-extension have been known; even the elastic extension has been applied by Brodie, but the methodical application of the treatment is due to Dr. Davis, and were it not for him the profession would have known nothing about it."—*American Medical Times, Vol.* 2, *No.* 17, *page* 278.

